# A comprehensive account of SARS-CoV-2 genome structure, incurred mutations, lineages and COVID-19 vaccination program

**DOI:** 10.2217/fvl-2021-0277

**Published:** 2022-06-16

**Authors:** Vijay Rani Rajpal, Shashi Sharma, Deepmala Sehgal, Apekshita Singh, Avinash Kumar, Samantha Vaishnavi, Mugdha Tiwari, Hemal Bhalla, Shailendra Goel, Soom Nath Raina

**Affiliations:** ^1^Hansraj College, University of Delhi, Delhi, 110007, India; ^2^Virology Division, Defence Research and Development Establishment, Gwalior, Madhya Pradesh, 474002, India; ^3^International Maize & Wheat Improvement Center (CIMMYT) Carretera México-Veracruz Km. 45, El Batán, Texcoco, 56237, México; ^4^Amity Institute of Biotechnology, Amity University Uttar Pradesh, Sector 125, Noida, Uttar Pradesh, India; ^5^Department of Botany, Vinoba Bhave University, Hazaribag, Jharkhand, 825319, India; ^6^Department of Botany, Central University of Jammu, Rahya Suchani (Bagla), Distt. Samba, Jammu and Kashmir, 181143, India; ^7^ICMR-National Institute of Occupational Health (ICMR-NIOH), Meghaninagar, Ahmedabad, 380016, India; ^8^Department of Botany, University of Delhi, Delhi, 110007, India

**Keywords:** corona viruses, COVID-19, genome structure, lineages, novel mutations, resistance to vaccines, SARS-CoV-2, vaccines

## Abstract

This review collates information on the onset of COVID-19, SARS-CoV-2 genome architecture, emergence of novel viral lineages that drove multiple waves of infection around the world and standard and fast track development of vaccines. With the passage of time, the continuously evolving SARS-CoV-2 has acquired an expanded mutational landscape. The functional characterization of spike protein mutations, the primary target of diagnostics, therapeutics and vaccines has revealed increased transmission, pathogenesis and immune escape potential in the variant lineages of the virus. The incurred mutations have also resulted in substantial viral neutralization escape to vaccines, monoclonal, polyclonal and convalescent antibodies presently in use. The present situation suggests the need for development of precise next-generation vaccines and therapeutics by targeting the more conservative genomic viral regions for providing adequate protection.

## Introduction

Several infectious viral disease outbreaks like Influenza A (H1N1), SARS, Middle East respiratory syndrome (MERS), Ebola and Zika have struck mankind in the past few decades. The present global health crisis, COVID-19 caused by SARS-CoV-2 has been the most catastrophic pandemic to hit the world during the last century. It has gripped the globe with its ubiquitous presence and has hit rich and poor nations alike across the continents.

COVID-19 pandemic management has presented an arduous task before the governments, policymakers and scientific community around the globe. Governments all over the world have taken strict measures to contain the transmission of the virus by imposing travel restrictions, recommending social distancing, compulsory wearing of masks and suspending educational and economic activities [1,2]. The scientific community around the globe, on the other hand, has shown commendable tenacity and has risen to this unprecedented situation to make some remarkable achievements in the form of developing diagnostics, therapeutic procedures and vaccines [3,4]. Nations worldwide have aimed to achieve exigent and safe herd immunity in human populations, to beat this monstrous pandemic as soon as possible. COVID-19 causes pneumonia in the infected people that can advance to SARS (severe acute respiratory syndrome), multiple organ failure and death. According to the WHO, globally there have been 412,351,279 confirmed cases of COVID-19 including 5,821,004 deaths across 240 nations of the world as of 15 February 2022 [5].

## Corona viruses & SARS-CoV-2

Coronaviruses (CoVs) belonging to the family Coronaviridae of the order Nidovirales are large (genome size: 27–32 Kb), non-segmented, enveloped, positive single-strand RNA viruses that have been responsible for many respiratory disease outbreaks including SARS and MERS . They are named ‘corona viruses’ due to their distinctive protein spikes. Depending upon their host infectivity, they are categorized into 4 genera [6]. While alpha and beta coronaviruses (alphaCoVs and betaCoVs) infect only mammals, gamma coronaviruses (gammaCoVs) infect avian species and delta coronaviruses (deltaCoVs) infect both mammals and avian species. BetaCoV genus is further divided into four sub-genera/lineages A, B, C and D [7], and includes the largest known RNA viruses.

Starting from the mid-1960s to till date, seven human coronaviruses (HCoVs) have been identified. While HCoV-OC43 and HCoV-HKU1 are betaCoVs belonging to lineage A, HCoV-229E and HC0V-NL63 are alphaCoVs. These CoVs have been observed to cause the common cold and mild upper respiratory tract infections. On the other hand, betaCoVs of the B and C lineages including SARS-CoV and MERS-CoV are more virulent viruses and cause severe respiratory problems that can lead to epidemics. An account of different HCoVs has been given in Table 1.

**Table 1.** Human Corona viruses: prevalence, infection and receptors.

Corona virus	Year	Natural host	Intermediate host	Illness level	People infected	Fatality rate	Receptors	Study (year)	Ref.
HCov-229E	Mid 1960s	Bat	Dromedary camels	Mild	15–30%	NA	Human aminopeptidase N (hAPN)	Liu *et al.* (2021)	[89]
HCov-OC43	Mid 1960s	Rat	Cattle	Mild	15–30%	NA	O-acetylated sialic acid	Liu *et al.* (2021)	[89]
HCov-NL63	2004	Bat	Unidentified	Mild	15–30%	NA	ACE2	Liu *et al.* (2021)	[89]
HCov-HKU1	2005	Rat	Unidentified	Mild	15–30%	NA	O-acetylated sialic acid	Liu *et al.* (2021)	[89]
SARS-CoV	2002–2003	Bat	Palm civet cat	Acute, fatal	∼8,000	∼10%	ACE2	Peiris *et al.* (2003)	[90]
MERS-CoV	Since 2012	Bat	Dromedary camels	Acute, fatal	∼1,700	∼36%	di-peptidyl peptidase 4 (DPP4)	Zaki *et al.* (2012)	[91]
SARS-CoV-2	Since Dec. 2019	Bat	Pangolin	Acute, fatal	More than 400 million as on 15 February 2022	3–5%	ACE2 and transmembrane serine protease 2 (TMPSSR2)	Liu *et al.* (2021), WHO (2022)	[5, 89]

SARS-CoV-2 is a spherical-shaped, beta coronavirus belonging to lineage B (β-B coronavirus) and contains a single-stranded, positive-sense RNA (+ssRNA) and is one of the largest RNA viruses known. The virus surfaced in Wuhan city, China in human beings in December 2019 and was initially termed 2019-nCoV (2019, novel corona virus) due to its larger similarity with coronaviruses [8]. Later investigations revealed 82% total genomic similarity and 96 and 89.6% sequence similarity in the envelope and nucleocapsid proteins, respectively between SARS-CoV-2 and SARS coronavirus (SARS-CoV) [8]. Additionally, SARS-CoV-2 and SARS-CoV both use the same ACE2 receptors for host entry, hence nCoV was renamed SARS-CoV-2 by WHO [5].

## Origin & spread

Genomic studies of coronaviruses have revealed that alphaCoVs and betaCoVs have most probably originated from bats and rodents, respectively. DeltaCoVs and gammaCoVs, on the other hand, seem to have originated from the avian species. The SARS-CoV-2 virions are 50–200 nm in diameter and their genome shows 96% nucleotide similarity to bat CoV, 91% to pangolin CoV, 80 % to SARS-CoV-1, 55% to MERS, and 50% to common influenza CoV [9].

The virus has initially been reported to have a connection with Huanan Seafood Market in Wuhan city, China, where it presumably originated by a zoonotic spill from bats to humans with pangolins as intermediate hosts. The viral genome has undergone many mutations to jump species and infect a new mammalian host [8]. After the first COVID-19 case was reported from Wuhan, China in December 2019, the disease showed a rapid spread in different parts of the world and within no time on 17 January 2020, it was reported from the Western Pacific; on 24 January 2020, from the Americas; on 26 January 2020, from the South East Asia; on 28 January 2020, from Europe; on 8 February 2020, from Eastern Mediterranean and on 25 February 2020, from Africa [5]. As on 15 February 2022, among the ten worst COVID-19-affected nations, USA (770,578,120) leads with maximum number of confirmed cases followed by India (42,692,943), Brazil (27,479,963), France (21,127,104) and the UK (18,348,033) [5]. With time, SARS-CoV-2 has shown significant evolution that is reflected in the form of induction of many novel mutations and emergence of novel genetic lineages with increased transmission/pathogenesis and immune evasion and spread to 240 countries and territories around the world [5].

## SARS-CoV-2 genome structure & functions

**Figure 1.**
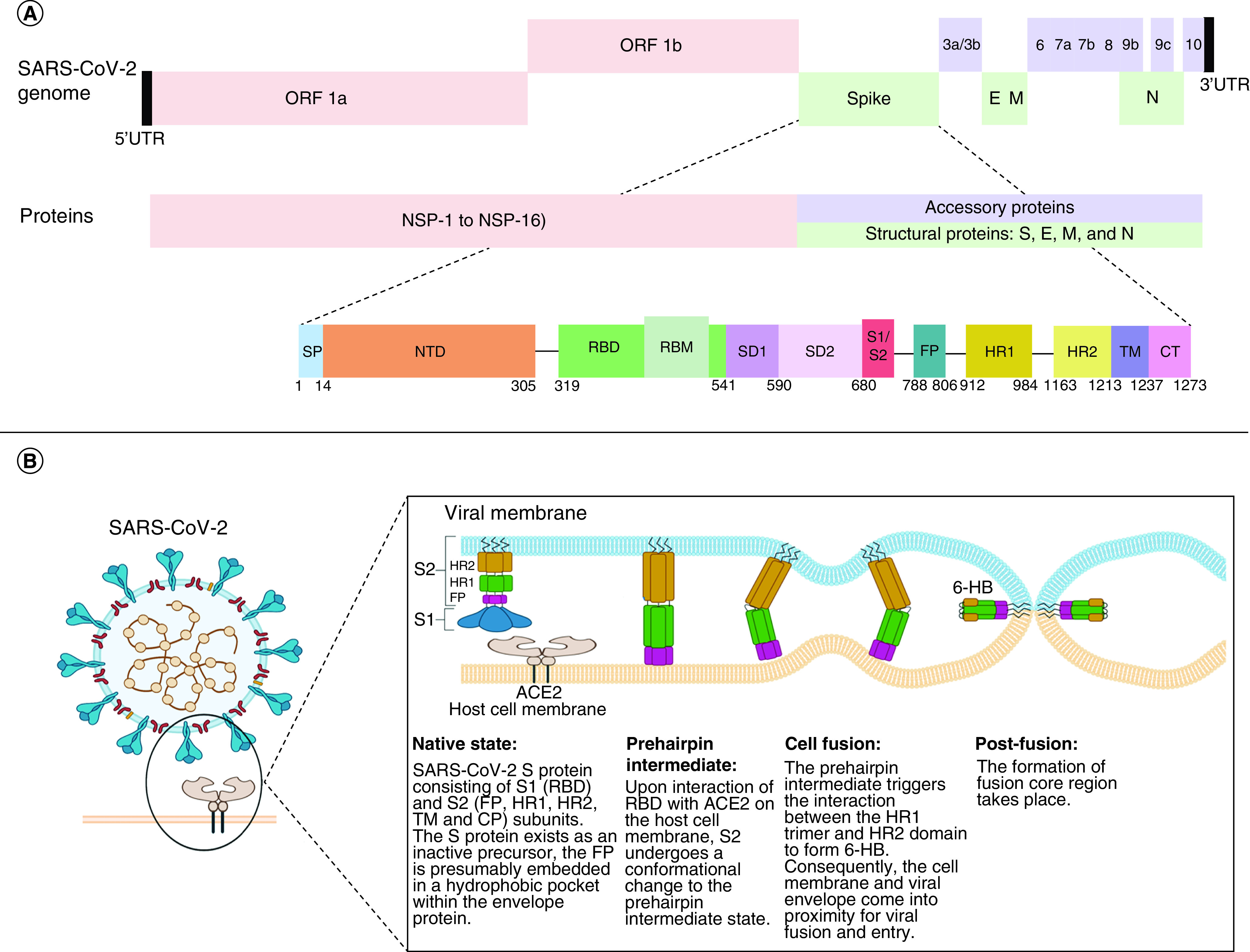
Structure and transmission of SARS-CoV-2. **(A)** Structure of SARS-CoV-2 genome and its spike protein. **(B)** Role of spike protein during the viral entry into the host. NSP: Nonstructural protein; NTD: N-terminal domain; RBD: Receptor-binding domain.

SARS-CoV-2 Wuhan-Hu-1 isolate sequencing (GenBank: MN908947.3) has revealed a genome size of 29,903 bp. The viral genome (+ssRNA) contains six major (ORF1a, ORF1b, S, M, E and N) and many accessory ORFs (ORF 3a/b, ORF6, ORF7a/b, ORF8, ORF9b/c and ORF10; Figure 1A). ORF1a and ORF1b account for two-thirds of the viral genome. ORFs encode 29 proteins that fall into two categories: nonstructural and structural proteins [10].

### Nonstructural proteins (NSPs)

The largest viral Orf1a/b represents two-thirds of the viral genome and has overlapping ORFs that produce two polyproteins pp1a and pp1ab. Two viral-encoded proteases cleave these precursor polyproteins into 16 nonstructural proteins (nsp1–16). NSPs include an RNA-dependent RNA polymerase (RdRp), RNA binding proteins, associated co-factors for replication, an exonuclease for proofreading, 3-chymotrypsin-like protease, papain-like protease, helicase, 3′-5′ endonuclease, N7 and 2′-O-ribose methyltransferase, and others encoded by the ORF region. NSPs enable viral replication, translation and assembly. They modulate transcriptional regulation, helicase activity and counter the antiviral response, and contribute towards virus inflicted pathogenesis [11].

### Structural proteins

The remaining viral genome encodes the structural proteins including spike, membrane-bound, nucleocapsid and envelope proteins [12]. Capsid-forming structural proteins are responsible for host recognition, membrane fusion, viral entry and release of virus particles [11,12].

#### Spike (S) protein

S protein is a large clove-shaped glycoprotein that covers the surface of SARS-CoV-2. S protein recognizes and binds to the ACE2 host cell surface receptors and mediates virus entry into the host. The spikes are coated with polysaccharides that serve a camouflage function and help the virus evade the immune system of the host to facilitate entry. Besides, the spike protein also determines the viral host range, tissue tropism and host immune response.

#### Nucleocapsid (N) protein

N protein contains approximately 140 amino acids long RNA-binding domain present in its core that helps it to bind to viral RNA. These structural proteins are associated with the envelope and help in virus assembly [13].

#### Membrane-bound (M) protein

Transmembrane glycoproteins are the most abundant structural proteins and function for the assembly of the virus. M protein is 222 amino acids long and along with envelope and N proteins helps in RNA packaging and virus assembly [13].

#### Envelope (E ) protein

The E protein is the smallest (75 amino acids) of all the structural proteins and functions for morphogenesis, assembly and release of virions [13].

Besides the above-mentioned proteins, SARS-CoV-2 encodes 8 accessory proteins derived from accessory ORFs 3a, 3b, 6, 7a, 7b, 8, 9b and 10 that are distributed among the structural genes (Figure 1A).

### Structure of S protein

S protein is encoded by the *S* gene that is 3,822 bp long and located in the 3rd ORF [12] with a molecular mass of 180–200 kDa, S protein consists of 1273 amino acids. At the N-terminus, amino acids 1–13 form a signal peptide (SP) that is followed by S1 (13-541 residues) and S2 subunit (788-1273 residues) (Figure 1A) [14].

#### S1 Subunit

S protein subunit S1 is composed of 672 amino acids and is organized into the N-terminal domain (NTD; 13-305) followed by the receptor-binding domain (RBD; 319–541 residues) (Figure 1A) [14]. RBD is a protein domain of 222 residues and 25 kDa molecular mass. The receptor-binding motif (RBM; 438–506 residues) that is present within the RBD forms a concave surface that engages with the outer claw-like structure of host ACE2 receptors. RBM shows sequence diversity among coronaviruses resulting in different receptor specificities.

#### S2 Subunit

Spike protein S2 subunit is composed of 485 amino acids that get organized into many domains and subdomains [14]. A proteolytic cleavage site called the furin-cleavage site is present between S1 and S2 subunits and is represented by a PRRA sequence motif [15]. A second proteolytic cleavage site in the S2 subunit is called the ‘S2’, is located upstream of the fusion peptide. Both these cleavage sites play a role in the viral entry. S2 subunit includes five major domains (Figure 1A). Fusion peptide (FP; 788–806 residues) is largely composed of glycine and alanine residues. FP holds to the host membrane when S protein takes up a pre-hairpin conformation [12] and brings about membrane fusion which is essential for viral entry. Heptapeptide repeat sequence 1 (HR1; 912–984 residues) and HR2 (1163-1213 residues) are composed of a repeat unit with monomer HPPHCPC containing a hydrophobic residue H, a polar or hydrophilic residue P and a charged residue C. HR2 is located at the N-terminus of the downstream transmembrane (TM; 1213–1237 residues) domain that culminates into the last domain cytoplasmic tail (CT; 1237-1273) of the spike protein. HR1 and HR2 interact to form a 6-helical bundle (6-HB) (Figure 1B) which is essentially required for viral fusion and entry, and the downstream TM domain anchors the S protein to the viral membrane during the entry process.

### Functions of S protein in the life cycle of SARS-CoV-2

Host receptor identification is the first step in the viral pathogenic invasion. In contrast to MERS-CoV, which recognizes dipeptidyl peptidase-4 (DPP4) as its receptor, SARS-CoV-2 recognizes ACE2 host receptors. ACE2 is a protein that is ubiquitously present on cell membranes and is abundant in the lungs, heart, kidney and intestines [16]. Host receptor binding is mediated by viral S protein. S protein remains inactive in its native state and during infection, it gets activated with the help of proteases of the host cell membrane using a transmembrane protease serine 2 (TMPRSS2) as a primer [9]. Upon receptor recognition, the host proteases cleave S protein into its subunits S1 and S2 at a furin-cleavage site [17]. S1 subunit is responsible for host cell receptor recognition, receptor interactions and binding, while the S2 domain brings about viral and host cell membrane fusion and viral entry into the host.

#### Receptor identification & binding

RBD present in the S1 subunit of S protein is the recognition site that binds to the peptidase domain (PD) of the ACE2 receptor of the host. Within the RBD, RBM is the precise region that interacts with the claw-like structure of PD of ACE2 receptors [18]. Engagement takes place between the N-terminal α1/α2 helices of ACE2 and the RBM motif. S protein functions as a conformational machine and takes up various conformations during the infection cycle and transits from closed to open conformation to expose its RBD. RBD of S1 undergoes hinge-like conformational movements to engage with the ACE2 host receptor [15]. The movements either expose or hide the receptor-binding regions and are termed as ‘up’ (receptor accessible) or ‘down’ (receptor inaccessible) conformations. RBD binding to cell surface receptor ACE2 prompts S1 to dissociate from ACE2 and this sets the stage for S2 to come into action for membrane fusion [17]. The function of the S1 subunit is to mediate receptor recognition and viral binding to the host membrane.

#### Membrane fusion & viral entry

While the RBD of the S1 subunit gets involved in molecular interactions with PD to bring about its binding to ACE2 on the host cell membrane, the S2 subunit is further exposed for cleavage by the host protease which is essential for viral infection [19]. The binding of S1 RBD to host membrane ACE2 brings about a conformational change in S2. The sequence of events begins with the first FP getting inserted into the target cell membrane. This allows the HR1 domain to transition from a metastable pre-fusion/native state to take up an intermediate short-lived pre-hairpin coil conformation. After this, HR1 and HR2 interact to form a six helical bundle (6-HB) that brings the viral and host cell membrane closer for membrane fusion and viral entry and sets the transition to metastable post-fusion conformation (Figure 1B). Recognition of the pre-fusion state is crucial to mounting an effective immune response and the transition between the conformations is genetically regulated. During this process, a homo-trimeric assembly of HR1 exposes its conserved hydrophobic groups for binding to HR2. During the post-fusion phase also, strong HR1–HR2 domains take place and form a fusion core region of the post-fusion conformation (Figure 1B) [14].

#### Virus multiplication

Following viral entry, genomic RNA of SARS-CoV-2 is released into the host cell cytoplasm where it replicates and also gets co-translated by the host machinery. Sixteen non-structural proteins and 4 structural proteins N, E, S and M get translated from ORF1a and ORF1ab and four structural genes, respectively. The nucleocapsid is formed by the association of the synthesized cytoplasmic N protein with the positive-sense RNA. Mature virions are formed when S, M, E and other viral proteins along with nucleocapsid are finally assembled into progeny viruses and then the mature virions are released from the host cell completing one life cycle.

## Variant genetic lineages of SARS-CoV-2

Viruses evolve with time as they move through humans or animals by acquiring single- or double-nucleotide changes in their genetic code. Multiple genetic lineages of SARS-CoV-2 have emerged globally due to the accumulation of novel mutations that have imparted an increase in transmissibility, pathogenesis or resistance to neutralization by antibodies to the virus. A US government SARS-CoV-2 interagency group (SIG), the European center for disease prevention and control (ECDC) and the WHO have developed lineage classification schemes that define SARS-CoV-2 variants either as variants of concern (VOCs), variants of interest (VOIs), variants under monitoring/being monitored (VUMs/VBMs) or variants of high consequences (VOHCs). Three nomenclature systems were established by the Global initiative on sharing all influenza data (GISAID), Nextstrain and Phylogenetic Assignment of Named Global Outbreak Lineages (PANGO) for naming and tracking SARS-CoV-2 genetic lineages [20–23].

Naming and characterization of these variants have helped in the global monitoring of the COVID-19 pandemic. Recently, the existing nomenclature systems have been modified by the WHO to be replaced by the use of Greek letters such as Alpha, Beta, Gamma, Delta, Omicron and others, to denote SARS-CoV-2 genetic lineages [21]. Further, there are differences in the labeling of lineages as VOCs or VOIs by the WHO, the ECDC and the CDC. While CDC recognizes only variants Delta (B.1.617.2 and AY lineages) and Omicron (B.1.1.529 and BA lineages) as VOCs and lists all other lineages as VBMs, no lineages are currently listed as VOIs and VOHC [21]. The WHO, on the other hand, recognizes five VOCs as Alpha (B.1.1.7, UK variant), Beta (B.1.351, South African variant), Gamma (P.1, Brazilian variant), Delta (B.1.617 and AY lineages) and Omicron (B.1.1.529 and BA lineages); two VOIs as Lambda (C.37) and Mu (B.1.621); and three lineages (B.1.1.318, C.1.2 and B.1.640) as VUMs. ECDC placement of lineages under VOCs, VOIs and VUMs is almost similar to WHO.

On 14 December 2020, a variant lineage SARS-CoV-2 VOC 202012/01 was reported by the UK to the WHO. It was defined by 23 nucleotide substitutions with multiple spike protein mutations (del69-70, del144, N501Y, A570D, D614G, P681H, T716I, S982A and D1118H) as well as mutations in other genomic regions [50]. The variant known as 501Y.V1 belongs to Nextstrain clade 20B, GISAID clade GR [20,21] and lineage B.1.1.7 and is now known as Alpha strain. The evidence has suggested that the Alpha variant has increased transmissibility. Another VOC was detected in South Africa, in December 2020 belonging to 501.V2 or B.1.351 lineage which is now designated as Beta strain [20,21]. The variant showed 9 mutations in the spike region (L18F, D80A, D215G, R246I, K417N, E484K, N501Y, D614G and A701V). It has gained increased virulence and transmission due to three RBD region mutations E484K + K417N + N501Y that increase ACE2 receptor-binding efficiency [24].

The third VOC, the Gamma variant also designated as P.1 or GR/501Y.V3 belongs to lineage B.1.1.28 and was identified in December 2020 in Brazil. This variant harbors ten mutations in the spike protein (L18F, T20N, P26S, D138Y, R190S, H655Y, T1027I V1176, K417T, E484K and N501Y). Similar to the Beta variant, three mutations (L18F, K417N and E484K) are located in the RBD. Notably, this variant may have reduced neutralization by monoclonal antibody therapies, convalescent sera and post-vaccination sera [25]. A deadly wave of infections was caused by another VOC, B.1.617.2 also referred to as the Delta variant, initially identified in December 2020 in India in April 2021. The B.1.617.2 variant harbors ten mutations (T19R, G142D, 156del, 157del, R158G, L452R, T478K, D614G, P681R and D950N) in the spike protein. Many of these mutations may affect immune responses directed toward the key antigenic regions of RBD [26]. Due to P681R mutation at the S1–S2 cleavage site, this strain may have increased replication, leading to higher viral loads and increased transmission [27]. In November 2021, the virus strain of lineage B.1.1.529 designated as the Omicron variant was first identified in South Africa. It soon emerged as a VOC due to its rapid transmission with more than 30 mutations in the spike protein of the virus [28]. Some of the major mutations recognized are N211del/L212I, Y145del, Y144del, Y143del, G142D, T95I, V70del, H69del, A67V in its NTD; Y505H, N501Y, Q498R, G496S, Q493R, E484A, T478K, S477N, G446S, N440K, K417N, S375F, S373P, S371L, G339D in the RBD; D796Y in the FP and L981F, N969K, Q954H in the HR1 of the spike region. The virus strain, although less severe in terms of hospitalization and deaths, proved 2.8-times more infective than the Delta variant and evaded neutralizing antibodies due to combinations of mutations [29].

According to the WHO, two listed VOIs are variants Mu and Lambda. Variant Lambda (C.37) belonging to GR/452Q.V1 clade was first detected in Peru in December 2020 and was designated as a VOI in June 2021 due to its increased presence. Another variant Mu (B.1.621) belonging to the GH clade designated as a VOI in August 2021, was first detected in Columbia in January 2021.

There are several other variants based on lineages with varied spike mutations that are under circulation in a restricted community and are still not named but are being monitored (VUMs) based on scientific observations and evidence. Variants B.1.1.318 (GR clade) and B.1.640 (GH/490R) were detected in multiple countries in January and September 2021, and designated as VUMs in June and November 2021, respectively.

## Novel SARS-CoV-2 mutations

Since its emergence in 2019, as many as 29,208 single mutations have been documented from 33,344,784 SARS-CoV-2 genomes [30], as of 31 January 2022. Several SARS-CoV-2 genes including *S, N* and *NSP12* (RdRP) have shown a high mutational range [31], though mutations have also been reported from other genomic regions, including ORFs 7b, 8, 10, nsp3 and others [31–39]. Hassan *et al.* [36] detected 128 single and 35 co-occurring mutations in ORF10 protein with a significant effect on pathogenicity. They also reported decreased stability of ORF10 because of missense mutations. Further, a shift in solubility and antigenicity between SARS-CoV-2 and Pangolin CoV show that 97.37% similarity in amino acid sequences was attributed to substructural differences between two ORF10 proteins [33]. Demographically dependent mutations and a periodic emergence of dominant mutations have been reported in different countries with the incidence of highly frequent mutations in ORF7b and ORF8 [35]. Mutations R203K and G204R in the N protein and Q27stop mutation in ORF8 have been associated with high viral transmission and immune evasion [7,37].

### Functionally characterized SARS-CoV-2 spike mutations

**Figure 2.**
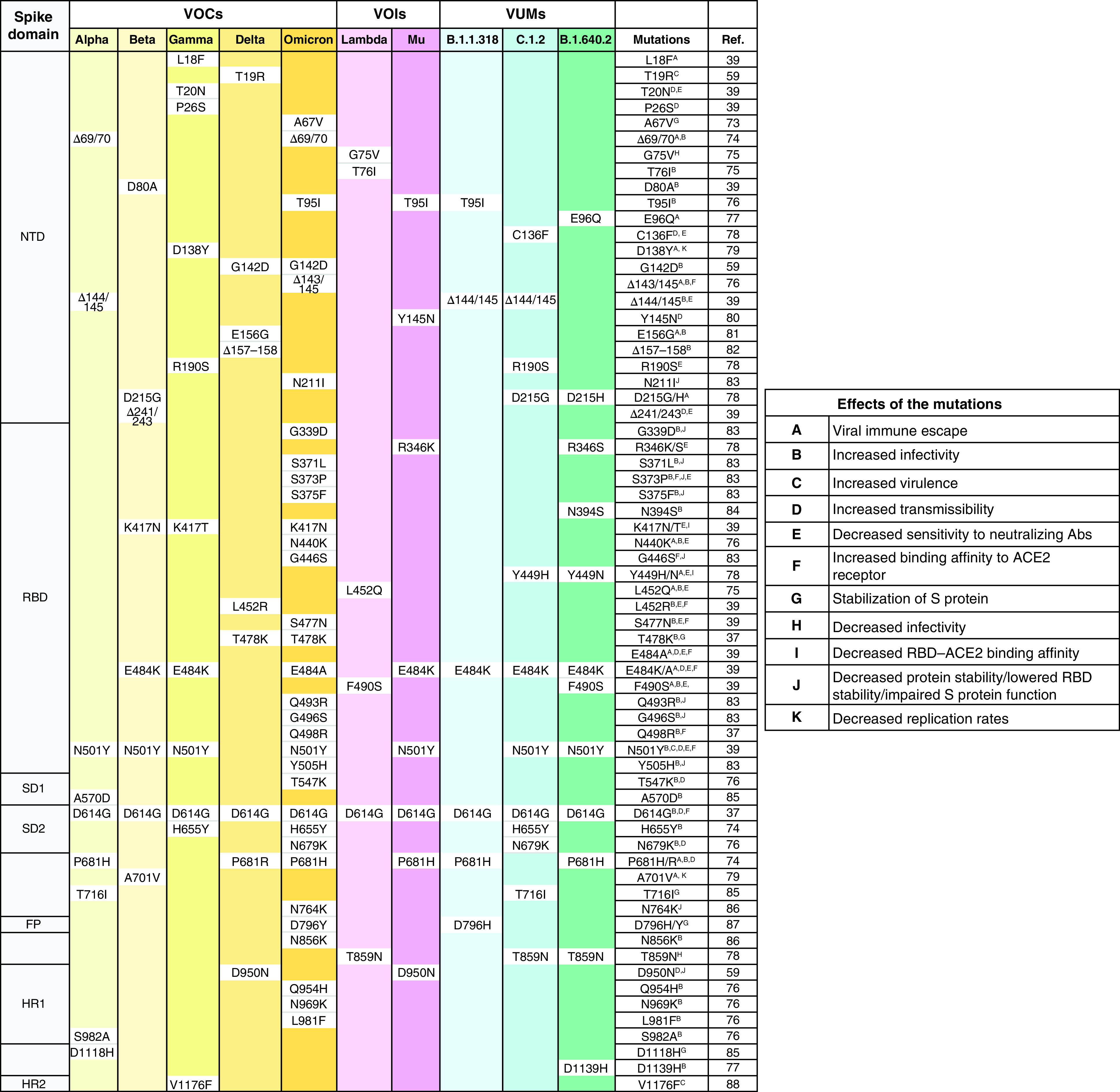
Functionally characterized mutations in various variants of concern, variants of interest and variants under monitoring of SARS-CoV-2. RBD: Receptor-binding domain; VOC: Variant of concern; VOI: Variant of interest; VUM: Variant under monitoring.

**Figure 3.**
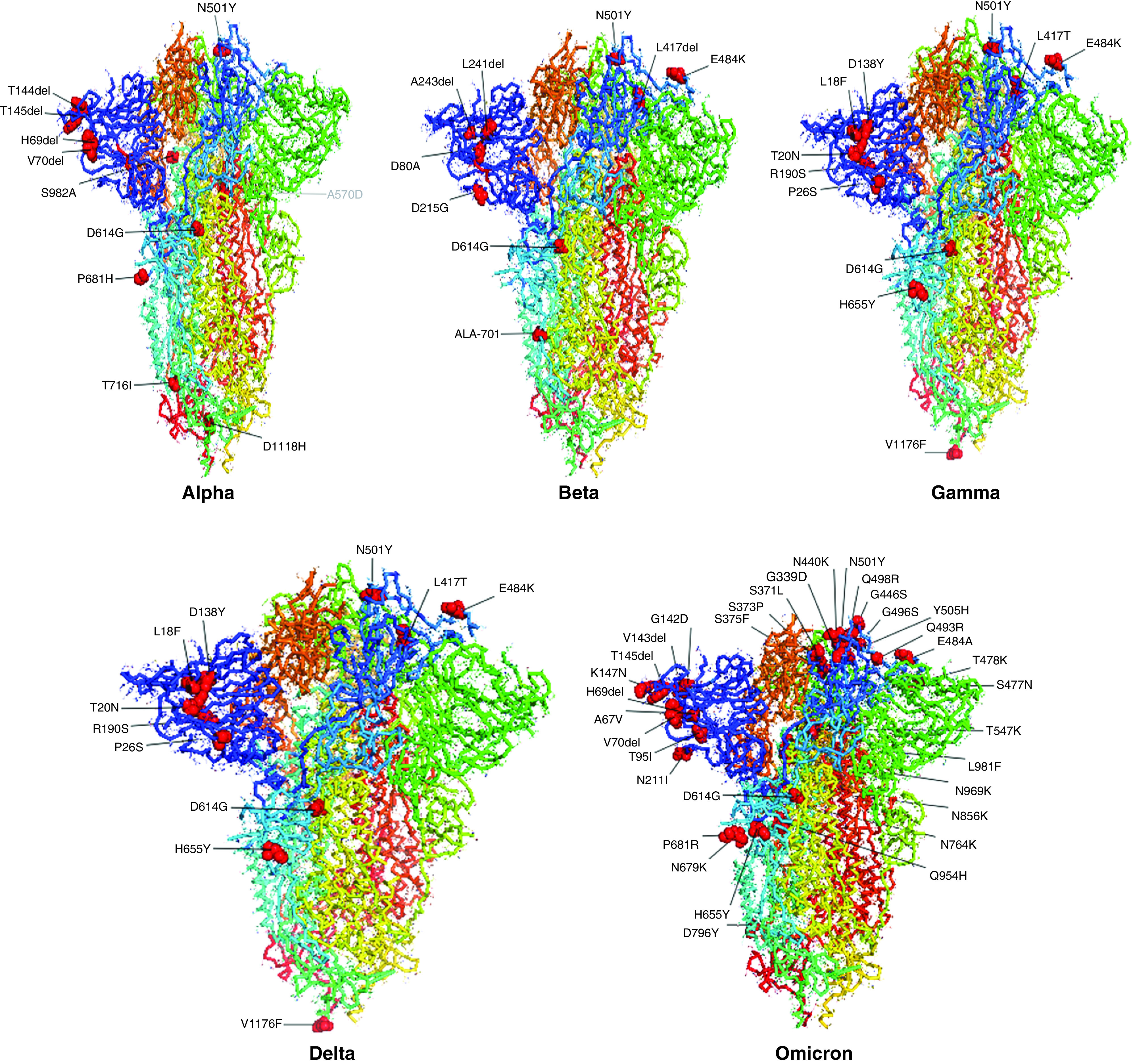
Functionally characterized mutations denoted on full spike protein trimer (Protein Data Bank: 6ZGE) in various variants of concern of SARS-CoV-2.

**Figure 4.**
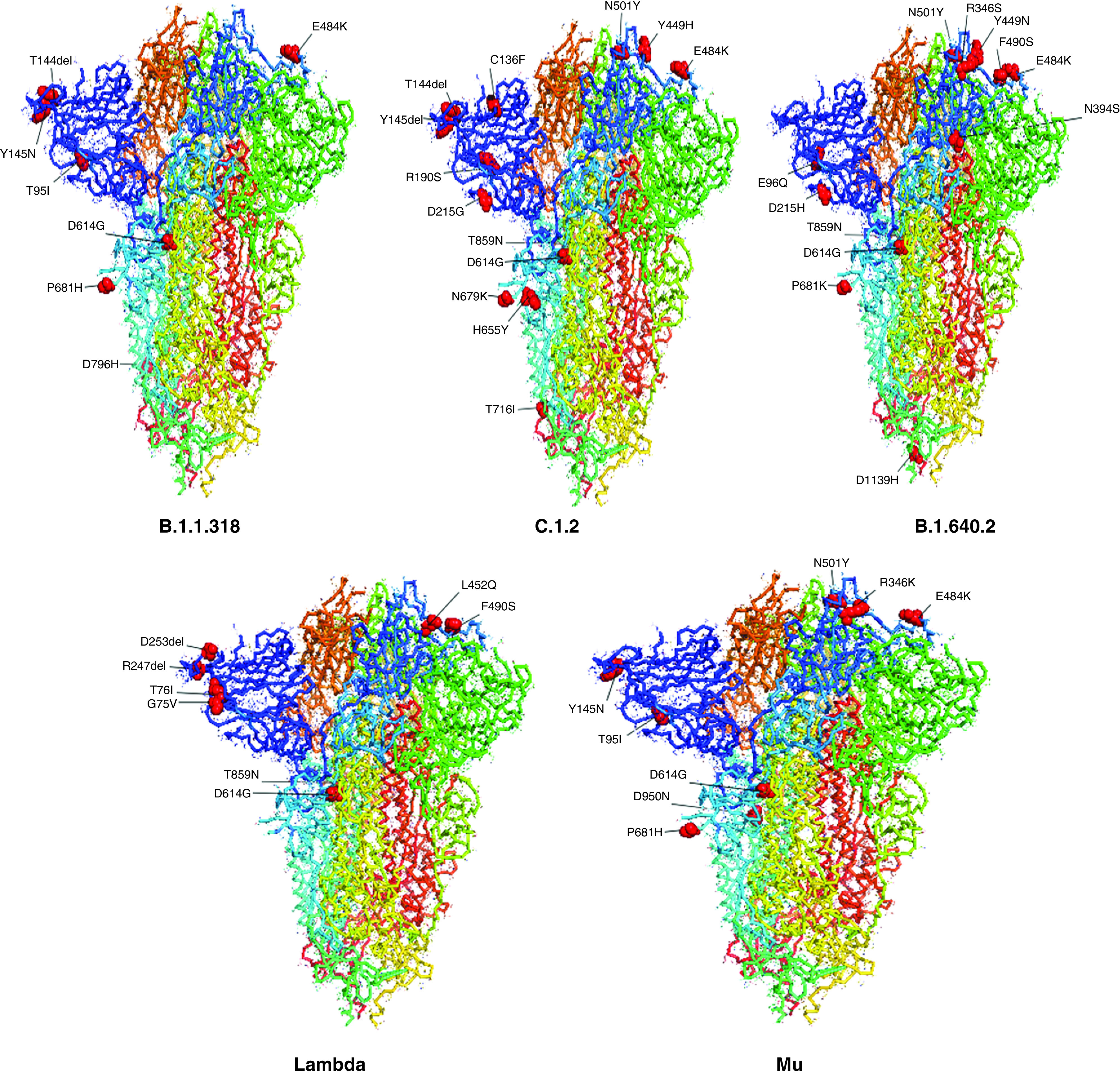
Functionally characterized mutations denoted on full spike protein trimer (Protein Data Bank: 6ZGE) in various variants of interest and variants under monitoring of SARS-CoV-2.

S protein has shown demography-specific evolution as revealed by Hassan *et al.* [34], who compared S protein variants from all the continents and detected their highest presence in South Africa. More than 6000 mutations have accumulated in the spike region in the last two years, and these are of particular concern because of the role spike protein plays in host pathogenesis, diagnostics, therapeutics and vaccine development [32,37,39,40]. Close to eighty of these documented spike mutations have been associated with one or more phenotypic effects in various WHO-listed VOCs, VOIs and VUMs (Figure 2). Most of these are substitution and deletion mutations with only a few insertions. Notably, the majority of these lie in the NTD (19) and RBD (25). Since it is the RBD that binds to the hACE2 receptor, many of the mutations in this domain such as S373P, N439K, G446S, L452R, Y453F, S477N, E484A, E484K and N501Y have enhanced the receptor-binding affinity. D614G, among the earliest and prominent mutations, evolved nearby and enhances receptor binding by smoother movement facilitated by the small glycine residue instead of the larger aspartate. Further, along with the antigenic epitope rich NTD supersite, the RBD also exhibits immunodominance because of less glycosylation and is thus the prime target for the immune response which has been shown to have reduced as a result of several mutations, such as L18F, Δ69/70, D138Y, Δ143/145, E156G and D215G in the NTD and V367F, K417N, N439K, N440K, Y449H, L452Q, E484K/A and F490S in the RBD. P681R mutation at the S1–S2 cleavage site has resulted in increased replication, leading to higher viral loads and increased transmission. The S2 region facilitates membrane fusion and sporadic mutations (T716I, D796H/Y, T859N, T859N and D1118H) in this region have been reported to promote the stabilization of the S protein. Together, these groups of mutations confer the virus with the distinct advantage of immune evasion, increased infectivity and transmissibility. The 20 most common RBD mutations in the analyzed lineages include E484A/K, G339D, G446S, G496S, K417N/T, L452R, N440K, N501Y, Q493R, Q498R, R346K, S371F/P, S373P, S375F, S477N, T478K and Y505H [30]. In Figures 2–4, we have tabulated and mapped the functionally characterized spike protein mutations in different VOCs, VOIs and VUMs of SARS-CoV-2 [30].

## Vaccines

Amidst the COVID-19 pandemic with its associated comorbidities and mortality, the only visible saviors for the human race have been the development of safe and efficacious vaccines.

Vaccines are prophylactic biological formulations, made of attenuated or dead forms of a pathogen, or any of its antigenic component(s) administered to stimulate an innate immune response for safeguarding against infections [41]. The details of vaccines developed using various approaches and that have completed phase III clinical trials and have been released for public use are presented in Table 2.

**Table 2.** Vaccines that have completed phase III clinical trials and approved for public use.

Vaccine	Developer	Type of vaccine	Target(s) of vaccine	Phase of clinical trial	Participants in phase III (n)	Emergency or full approval (countries authorized for public use)	Efficacy
Ad5-nCoV (Convidecia)	CanSino Biologics and Beijing Institute of Biotechnology of the Academy of Military Medical Sciences (China)	Recombinant adenovirus vector	Full-length S protein	Phase III	40,000	Emergency (China), in use by February 2021	65.7%
AZD1222 (Covishield in India) (ChAdOx1 nCoV-19)	AstraZeneca and Oxford university (UK)	Chimpanzee adenovirus vector	Full-length S protein	In use	30,000	Emergency (UK, India, Bangladesh, Argentina, Mexico, Dominican Republic and El Salvador)	81.0%
BBIBP-CorV	Sinopharm, Beijing Institute of Biological Products, Wuhan Institute of Biological Products (China)	Inactivated SARS-CoV-2	Complete SARS-CoV-2 virus	Phase III	48,000	Emergency (Egypt, Jordan), full (China, Bahrain and United Arab Emirates)	87.8%
Covaxin (BBV152)	Bharat Biotech, Indian Council of Medical Research (India)	Inactivated SARS-CoV-2	Complete SARS-CoV-2 virus	In use	25,800	Emergency (India), in use since January 2021	77.8%
CoronaVac	Sinovac Biotech Ltd. (Beijing, China)	Inactivated SARS-CoV-2	Complete SARS-CoV-2 virus	Phase III	33,620	Emergency (China, Bolivia, Indonesia and Turkey)	49.6%
Sputnik V (Gam-COVID-Vac)	Gamaleya National Center of Epidemiology and Microbiology (Moscow, Russia)	Adenovirus vectors rAd26-S and rAd5-S	Full-length S protein	In use	40,000	Emergency (Russia, Belarus, Argentina, Bolivia, Venezuela, Serbia, Guinea, Algeria and Palestine), in use in India and other countries	91.6%
EpiVacCorona	Vector Institute (Koltsovo, Russia)	Peptide antigens	Spike epitope	Phase III	40,000	Emergency (Russia and Turkmenistan)	79.0%
mRNA-1273	Moderna Therapeutics, National Institute of Allergy and Infectious Diseases, Biomedical Advanced Research and Development Authority (USA)	Modified messenger RNA	Full-length S protein with 2 proline substitutions (K986P and V987P)	In use	30,000	Emergency (USA, Canada, UK and Israel), full (EU, Norway, Iceland, Greenland and Switzerland)	94.1%
BNT162b2 (Tozinameran)	Pfizer (USA) and BioNTech (Germany)	Modified messenger RNA	Full-length S protein with 2 proline substitutions (K986P and V987P)	In use	43, 448	Emergency (UK Canada, Bahrain, US, Mexico, Kuwait, Singapore, Jordan, Oman, Costa Rica, Ecuador, Israel, Panama, Chile, Qatar, UAE, Argentina, Iraq, Colombia and Philippines), full (EU, Saudi Arabia, Switzerland, Norway, Iceland, Faroe Islands, Greenland, Serbia and Malaysia)	95%
Ad26.COV2.S	Johnson and Johnson, Beth Israel Deaconess Medical Center (USA)	Adenovirus vector	Full-length S protein with two furin cleavage site mutations (R682S, R685G) and two proline substitutions (K986P, V987P)	Phase III (in use)	40,000	Emergency (Canada, EU and South Africa)	95%
NVX-CoV2373	Novavax (MD, USA)	Recombinant SARS-CoV-2 (rSARS-CoV-2) nanoparticle with adjuvant	Full-length S protein with 682-QQAQ-685 mutations at the S1/S2 cleavage sites and two proline substitutions at residues K986P and V987P	Phase III (in use)	45,000	Emergency (Mexico)	90.4%
ZyCov-D	Zydus Cadila Healthcare Ltd., (Gujrat, India)	Genetically engineered DNA plasmid based vaccine expressing SARS-CoV-2 S protein	Full-length S protein	Phase III	26,000	Emergency (India)	66.6%
ZF2001	Anhui Zhifei Longcom Biopharmaceutical Co., Ltd. (China)	Recombinant subunit	RBD-dimer (residues 319–537 as tandem repeat)	Phase III	29,000	Emergency (China, Indonesia and Uzbekistan)	78.0%

### Standard & fast track development of a vaccine

**Figure 5.**
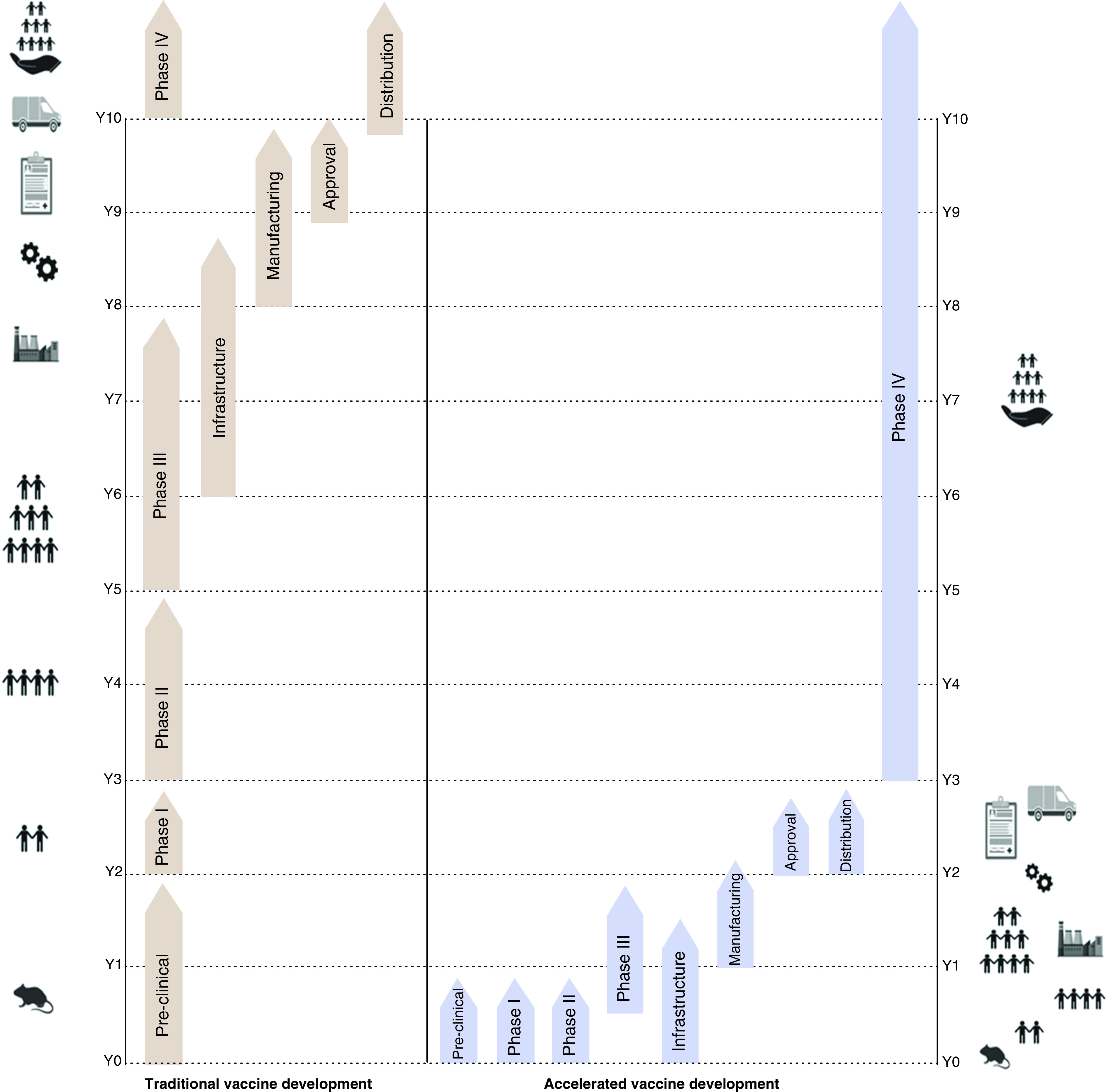
Timeline comparison between standard and fast-track development of vaccines.

Standard vaccine development is a long sequential process that can take from 5 years to even up to a decade (Figure 5). It involves making, testing in small batches using *in vitro* studies or animal models (*in vivo*), quality control, and finally three phases of clinical trials before approval for public use. The three phases of clinical trials involve; phase I human pharmacology studies, phase II therapeutic exploratory studies, and phase III safety studies. Phase 1 involves between 30 and 100 volunteers to confirm whether the vaccine triggers the expected immune response as was expected based on laboratory tests. Also, this phase allows the testing of different doses that can be administered safely to an individual. Phase II, which involves hundreds of volunteers, is conducted to study the best doses to use and the most common side effects. Phase II also investigates whether the vaccine can trigger a good immune response in a broader population. The final Phase III involves thousands of volunteers (20,000–40,000 individuals) to test the efficacy of the vaccine compared with a placebo which is a saline injection. Phase III is the most crucial stage of testing to secure approval from the regulators.

In a public health emergency, vaccine development can be fast-tracked. During the fast-track development of a vaccine, efficacy, quality and safety are not compromised but are embedded early in the process (Figure 5). After the declaration of the coronavirus pandemic a public health emergency (PHE), WHO validated the fast-track development of vaccines to halt the spread of COVID-19 around the world.

In the USA, the FDA has the authority to use its Emergency Use Authorization (EUA) policy during a public health emergency to permit the use and release of unapproved or off-label use of medical products and/or vaccines to treat or prevent serious or life-threatening diseases when no adequate, approved and available alternatives exist. In relation to the COVID-19 pandemic, on 4 February 2020, the Secretary of Health and Human Services declared the emergency use of FDA-regulated therapeutic products including fast-track development of vaccines [42]. The pharmaceutical companies Pfizer, Moderna and Johnson and Johnson vaccines were initially issued EUAs to produce COVID-19 vaccines [43,44].

In the EU, the European Medicines Agency (EMA) plays an important role in the development, scientific evaluation, approval and monitoring of vaccines. To fast-track the development of COVID-19 vaccines, EMA has made a COVID-19 EMA pandemic Task Force (COVID-ETF), whose main role is to help EU member states and the European Commission to take quick regulatory action on the development and authorization of vaccines for the prevention of COVID-19. In addition, COVID-ETF provides scientific support by extending collaboration with the clinical trials facilitation group (CTFG) to facilitate clinical trials conducted in the EU [45]. Under the fast-track development procedure, the timeline to release a vaccine has been reduced significantly from 10 years to 18 months. In Figure 5, a standard and fast-track vaccine development protocol has been compared.

### Vaccine platforms for COVID-19

When the whole world came to a standstill because of globe-wide lockdowns, the scientific community was on a mission to understand the perpetrator and to develop strategies to control it. Rapid sequencing efforts initiated in January 2020 revealed high sequence similarity of the SARS-CoV-2 virus with SARS-CoV [46]. Structural similarities between the two viruses were also reported based on cryo-electron microscopy. Both these developments gave a jump-start to the design of vaccines. Novel technologies such as nucleic acid-based and computationally designed nanoparticle-based methods have been used concomitantly with existing technologies for antigen design, antigen display and vaccine delivery into the human body to speed up the development of vaccines [41,47]. The COVID-19 vaccination program is underway in 184 countries of the world with about 10.5 billion doses already administered as on 15 February 2022 [48].

More than 200 vaccine candidates exist for SARS-CoV-2, many of which have come into the market for public use and others are in different stages of pre-clinical and clinical trials. Details of vaccines developed using various approaches, vaccines in phase III trials, vaccines that have completed trials and released for public use are presented in Table 2. Vaccines have been developed using various platforms such as protein subunits, recombinant subunits, adenovirus-based vectors, messenger RNA, or even inactivated whole SARS-CoV-2 virus. The majority of vaccines, however, are based on protein subunit (∼70) followed by non-replicating viral vectors (∼30) and viral RNA [49].

Knowledge gained from RBD-based vaccines against SARS-CoV and MERS-CoV has served as a blueprint for the design of the RBD-based SARS-CoV-2 vaccines and to date, most of the potent neutralizing antibodies (NAbs) to SARS-CoV-2 target the RBD [50]. SARS-CoV-2 NAbs bind to the RBD and block its attachment to ACE2, thus inhibiting virus attachment. Scientists have adopted various strategies to further increase the potency of RBD-based vaccines, including increasing antigen size or RBD di- or multimerization [51]. Apart from RBD, the N-terminal domain (NTD) of subunit S1 can be a potential vaccine target [46]. Initial results, however, have shown that NTD-based subunit vaccines might prove less effective than RBD-based vaccines because a lower neutralizing efficiency was observed in NAbs targeting NTD as compared with RBD-specific Nabs [46]. Peptides derived from HR1 or HR2 of the S2 subunit of SARS-CoV-2 have also been used as additional potential vaccine targets. Other proteins such as N, E and M proteins and nonstructural proteins such as NSP13 and NSP15 could serve as antigens but only to broaden the T-cell response and improve cross-protection because of their small ectodomains and small molecular sizes [46].

A major advantage of protein subunit vaccines is that they can be used on people with weakened immune systems since they do not display the full antigenic complexity of the virus. Additional ‘booster shots’, however, are required with these vaccines for continuous protection. A protein vaccine, NVX-CoV2373, made by a US-based biotech company Novavax contains a full-length, prefusion spike protein made using recombinant nanoparticle technology in combination with saponin-based immune system-activating adjuvant (a compound that enhances the magnitude and/or durability of the immune response to the protein antigen). The phase III trials began in the USA and Mexico on 28 December 2020 and it got approval for first emergency use in Mexico and later in many countries.

The vaccines BBIBP-CorV, Covaxin, CoronaVac and PiCoVacc based on inactivated SARS-CoV-2 virus have completed their phase III trials and have received emergency or full approval for use. These vaccines use attenuated (via heat or chemical treatments) SARS-CoV-2 virus enabling our immune system to recognize multiple viral proteins S, M, E, N or NSPs. However, such vaccines may revert to virulence in immunocompromised individuals. Of the three vaccines developed, BBIBP-CorV, developed by a Chinese company Sinopharm, has received approval for full public use in China, Bahrain and the United Arab Emirates in addition to emergency approval in Egypt and Jordan. Covaxin, India's indigenous COVID-19 vaccine made by Bharat Biotech in collaboration with the Indian Council of Medical Research (ICMR) and National Institute of Virology (NIV) completed phase III clinical trials in November 2020 and demonstrated 81% interim clinical efficacy in preventing COVID-19. WHO has approved its emergency use in many countries. In India alone, more than 810 million doses of Covaxin have been administered. Another potential candidate, PiCoVacc, based on purified inactivated virus has entered phase III trials. It has shown the induction of NAbs against SARS-CoV-2 in mice, rats and rhesus monkeys [52].

Viral vector vaccines have made use of non-SARS-CoV-2 viruses such as adeno or pox virus in dead or weakened form with genetic material from SARS-CoV-2. The advantage of adenovirus-based vaccines is that they do not require ultra-low temperatures for storage, which makes distribution easier. Four viral vector vaccines *viz.* Sputnik V, Ad5-nCoV, AZD1222 and Ad26.COV2.S have already completed phase III trials and have been approved for emergency use in many countries (Table 2). The Gamaleya National Centre of Epidemiology and Microbiology in Russia made the first viral vector vaccine called Sputnik V which used two strains of adenovirus, Ad26 and Ad5. Sputnik V was cleared for widespread use in Russia in August 2020, even before phase III trials were conducted. It was registered as the first COVID-19 vaccine on the market. CanSino Biologics, a Chinese company, made a viral vector vaccine called Ad5-nCoV. Though the vaccine was still technically in phase II of its clinical trial in June 2020, the Chinese government approved the vaccine for military use only. Phase III trials were conducted in Argentina, Pakistan, Russia, Mexico, Saudi Arabia and Chile, and reports in February 2021, revealed an efficacy of 65.7–91%.

The COVID-19 Oxford vaccine (AZD1222) is a collaborative effort between the University of Oxford and pharmaceutical company AstraZeneca. It uses chimpanzee adenovirus (common cold virus [ChAdOx1]) combined with the genetic sequence of the SARS-CoV-2 surface spike (S) protein. Between 23 April and 4 November 2020, 7,548 individuals from the UK and 4,088 from Brazil participated in an interim primary efficacy analysis [53]. After completion of phase III trials, AZD1222 was approved for emergency use in many countries including the UK, India, Bangladesh, Argentina, Mexico, Dominican Republic, El-Salvador and the EU. A fourth viral vector vaccine Ad26.COV2.S made by Johnson & Johnson also received approval for emergency use in many countries after a successful phase III trial.

Nucleic acid vaccines use DNA or mRNA to induce an immune response. These types of vaccines are simple to create and include a genetic code for the pathogenic antigen and a delivery method. A total of 24 candidate vaccines have been developed for COVID-19, which are under different stages of clinical development [54]. mRNA-based COVID-19 vaccines induce immunity to SARS-CoV-2 by encoding a prefusion stabilized spike (S) protein. In the past, mRNA vaccines development for flu, Zika, rabies and cytomegalovirus had been initiated but never entered the clinical trial. mRNA-1273, developed by Moderna Therapeutics in collaboration with three other US institutes, is the first mRNA vaccine that entered human trials on 16 March 2020. On 17 December 2020, this vaccine received approval from the FDA for emergency use in USA. Pfizer, one of the largest pharmaceutical companies in collaboration with German company BioNTech, made another mRNA-based vaccine called BNT162b2. The UK was the first to approve BNT162b2 for human use on 2 December 2020, and later it was also granted emergency approval by the FDA.

DNA vaccines involve delivering DNA plasmids encoding immunogenic antigens and a mammalian promoter [55]. They can be administered via intramuscular, subcutaneous, mucosal, or transdermal routes and these elicit both humoral as well as cellular immunity. Currently, there are three DNA vaccine candidates for COVID-19 which have reached phase III clinical trials *viz.* ZyCoV-D from Zydus Cadila (CTRI/2021/01/030416), Takara by Osaka University (NCT04655625) and INO-4800 from Inovio Pharmaceuticals (NCT04447781, NCT04642638 and ChiCTR2000040146) [56].

### Resistance to vaccines

Most of the neutralizing serum and therapeutic antibodies target the RBD region in S protein which, as discussed before, has accumulated many mutations conferring adaptive advantage to the virus. Many of the induced mutations affect the efficacy of the therapeutic monoclonal antibodies (mAbs), convalescent plasma and vaccines because of the evolution of variants that escape antibody binding and neutralization [57,58]. Several studies on vaccine effectiveness (VE) against existing variants and lineages have been conducted and have been extensively reviewed as well [37,59,60].

At the beginning of the rollout of the vaccination program, even though single-dose vaccinated individuals showed the presence of neutralizing antibody titers against the Alpha, Beta and Delta variants, it was realized that the titers were still not at desirable levels. Many reports emphasized the need for a second dose of vaccine, which then became a standard regime for vaccinations around the world. The neutralizing efficiency was found to increase considerably after the second dose. The two-dose effectiveness against the Delta variant was estimated to be 60 and 88% for the ChAdOX1 ncov-19 and the BNT162b2 vaccine, respectively [61].

Various vaccines have reportedly shown varying degrees of effectiveness against different variants. As compared with the Alpha variant, decreased neutralization titers were observed against Beta (16-fold and ninefold) and Delta (threefold and fivefold) with BNT162b2 and ChAdOX1 ncov-19 vaccines, respectively [59]. Individuals vaccinated with nvx-cov2373 displayed 85.6% efficacy against the Alpha variant. BNT162b2 vaccine evoked 1.7 to 6.0-fold reduced nAb activity against the Alpha variant, 2.1 to 5.1-fold against Gamma, and 1.4 to 3.0-fold against the Delta variant [59]. Sera from individuals vaccinated with the ChAdOX1 ncov-19 vaccine displayed reduced (ninefold and 2.5-fold) nAb activity against the Alpha pseudovirus. Similarly, a 6.5 to 10.4-fold reduced nAb activity was observed against the Beta pseudovirus [37]. The ChAdOX1 ncov-19 vaccine also exhibited reduced nAb production for Beta, Gamma and Delta lineages [59,62]. Similarly, a 2.1 to 3.3-fold reduction against Delta was also reported for the mRNA1273 vaccine [37]. The Sputnik V, BBIBP-CorV and CoronaVac vaccines also showed reduced VE against Alpha, Beta and Gamma variants [63,64].

The EMA and FDA-approved vaccination schedules for various countries initially included the administration of two doses (except a single dose in Ad26.COV2.S) of the same (homologous) vaccine. This is known as the primary vaccination schedule. With time, reports of waning immunity with even a two-dose vaccine regimen, as listed above, started pouring in. Concurrently, the world over, a fresh wave of infections was triggered by the Delta and then the Omicron variants and numerous cases of reinfection and vaccine breakthroughs were reported. This prompted approval of homologous (with the same vaccine as in the primary schedule) or heterologous (different vaccine than the primary vaccine) booster doses to achieve adequate protection [65].

The VE and neutralization assays in the homologous or heterologous prime-boosted individuals have been done in many countries and is reviewed in Netzl *et al.* [60]. The studies have reported a significant reduction in neutralization with almost all the tested vaccines in both setups, though the heterologous prime-boosted regime has shown better neutralization titers. For instance, the uninfected people who received two doses of BNT162b2 or CoronaVac vaccine within 1 month had 30-fold lower PRNT (50% plaque reduction neutralization test) antibody titers against the Omicron variant [66]. While a 6.4-fold reduction was found in those who received two doses of CoronaVac plus a booster dose of the homologous vaccine against Beta or Delta RBD binding assays [67]. Computer modeling and numerous laboratory neutralization studies for the Omicron variant have shown a substantial reduction in vaccine neutralization by 30 to 40-fold and a drop in neutralization by other vaccines like mRNA1273, Sputnik V, Ad26.Cov2.S, ChAdOx1 ncov-19 and BBIBP-CorV [68,69]. It was observed that the so-called ‘hybrid immunity’ (previous infection and vaccination) demonstrated significantly better neutralization to counter the new variant Omicron [59].

Cumulatively, the published reports have shown that VE and neutralization potential are significantly affected by novel mutations in circulating lineages of SARS-CoV-2. Five of the most significant RBD substitutions E484K, N501Y, S477N, K417N and T478K documented in Alpha, Beta, Gamma, Delta and Omicron variants affect both ACE2 binding efficacy and relative neutralization to multiple mAbs and immune sera from convalescent and vaccinated people [38,59,64,70–72]. In the wake of fast-evolving genetic lineages, it is, therefore, important to watch the long-term durability of this vaccine's boosted immunity concerning reinfections and breakthrough infections.

## Conclusion

Three major pathogenic outbreaks of coronaviruses have happened in the past two decades. The first SARS-CoV outbreak in 2002 infected over 8000 people and the second outbreak of MERS-CoV infected over 2,294 individuals. The third and the ongoing pandemic by SARS-CoV-2 has been the most devastating with close to 6 million deaths around the globe. Besides a hefty death toll, the COVID-19 pandemic has resulted in social and economic disruption and has caused the largest global recession. The COVID-19 pandemic has been penned down as the most expensive disaster ever in human history. Although, commendable fast-track rollout of SARS-CoV-2 vaccines was expected to be the panacea from the pandemic. However, vaccine breakthroughs and immune escapes have been brought about by the emergence and rapid spread of new strains with novel mutations. It is anticipated that the evolutionary interplay of mutations would eventually plateau to a highly transmissible but less virulent variants. However, till then the role of the scientific community in tackling the virus cannot be overstated. While on one hand, the dominant variants have to be kept in control through standard operating procedures, on the other hand, surveillance for tracking emerging novel mutations has to be an ongoing process. *In silico* investigations in predicting the possible changes in antigenic sites, their repercussions *vis-à-vis* epitope–paratope interactions and recognition of conserved viral domains among others are some of the current research concerns. The research leads obtained can be used in the final objective of the development of a ‘variant proof pan SARS-CoV-2 vaccine’ to control this evolving nemesis.

## Future perspective

Ongoing global immunization efforts and acquired immunity after previous SARS-CoV-2 infections are optimistically thought to bring the COVID-19 pandemic under control. It is anticipated that SARS-CoV-2 will eventually become endemic with seasonal recurrent epidemic peaks, although there are still many uncertainties that need some time to get resolved. The global inequities in vaccinations, for instance, restrict achieving herd immunity anytime soon in the future. Further, reports of the waning of acquired immunity against novel variants with significant changes in antigenicity, the peril of emergence of a super virus arising out of recombination between simultaneous infection of two different viral strains in a single individual represent even bigger challenges.

The further antigenic evolutionary discourse of the virus in terms of increased transmission/virulence/immune evasion amidst the selection pressure posed by the immunized population and the future viral–human equilibrium, though difficult to predict accurately, might take into account further dwelling upon additional attributes like pantropic properties of the virus, generation time, adaptive selection pressure and emergence of novel variants, zoonotic evolution and dependence on most of the existing key vaccines and therapeutic molecules on heavily mutated spike protein that has shown enormous structural plasticity, in the coming years.

The lessons learnt from previous multiple infection waves across the world caused by various fast-evolving genetic lineages of SARS-CoV-2, nevertheless, suggest that effective surveillance especially in immunocompromised patients and poorly vaccinated pockets of the world, equitable distribution of vaccines, watching the long-term durability of vaccine and/or infection acquired immunity related to reinfections and breakthrough infections, study of viral evolution in non-spike regions, identification of conserved non-spike viral regions to design additional vaccines and therapeutics, *in silico* studies based prediction of the possible changes in antigenic sites, technologies to tweak the existing vaccines to neutralize the novel variants and finally the development of a ‘variant proof pan SARS-CoV-2 vaccine’ will be some key strategies for effective COVID-19 management and to prevent the emergence of new pandemic patterns.

**Executive summary**

**SARS-CoV-2 & COVID-19**

- COVID-19 caused by SARS-CoV-2 has resulted in social and economic disruption and close to 6 million deaths around the globe.
- The virus surfaced in Wuhan city, China in human beings in December 2019, was initially termed as 2019-nCoV and later as SARS-CoV-2. SARS-CoV-2 genome shows significant nucleotide similarity to bat, pangolin, influenza SARS-CoV-1 and even MERS CoV.

**SARS-CoV-2 genome**

- The viral genome contains six major (ORF1a, ORF1b, S, M, E and N) and many accessory ORFs (ORF 3a/b, ORF6, ORF7a/b, ORF8, ORF9b/c and ORF10) that encode 29 nonstructural (NSPs) and structural proteins along with many accessory proteins. NSPs enable viral replication, translation and assembly, while capsid-forming structural proteins are responsible for host recognition, membrane fusion, viral entry and release of virus particles.

**Variant genetic lineages**

- The virus is continuously evolving and multiple genetic lineages have emerged globally. Accumulation of novel mutations in the genome has conferred an adaptive advantage to the virus in terms of increased transmissibility, virulence, pathogenesis and immune escape.
- Based on their transmission and pathogenesis capacities, variant lineages have been categorized by WHO as five variants of concern (VOCs) Alpha (B.1.1.7, UK variant), Beta (B.1.351, South African variant), Gamma (P.1, Brazilian variant), Delta (B.1.617 and AY lineages) and Omicron (B.1.1.529 and BA lineages); two variants of interest (VOIs) as Lambda (C.37) and Mu (B.1.621); and three lineages (B.1.1.318, C.1.2 and B.1.640) as variants under monitoring (VUMs).

**Spike protein & incurred mutations**

- The spike protein (S protein) plays an important role in viral inflicted pathogenesis and is involved in host cell receptor recognition, viral attachment and entry into the host. More than 6000 mutations have accumulated in the spike region in the last two years with several being characterized by increased transmission, pathogenesis and immune-evasion functions. This is perturbing as most of our existing diagnostics, therapeutics and vaccines are spike based.

**COVID-19 vaccination program**

- COVID-19 vaccination program, the largest in human history ever is underway in 184 countries of the world and as of 15 February 2022, about 10.5 billion doses have already been administered. Multiple vaccine types exist that are based on protein subunits, recombinant subunits, adenovirus-based vectors, messenger RNA, or even inactivated whole-SARS-CoV-2 virus.

**Vaccine efficacy & resistance to neutralization**

- Though vaccines have largely been a savior but with the passage of time, a significant reduction in vaccine efficacy and the neutralization potential of monoclonal, polyclonal and convalescent antibodies have been observed in two-dose vaccinated individuals which has led to the addition of homologous and heterologous boosters to the two-dose standard prime vaccination regime.
- Reports of significant reduction of neutralization of variant lineages even in boosted individuals in vaccine efficacy studies as compared with the original SARS-CoV-2 virus has rung the warning bell and the scientific community is keeping a close watch on the long-term durability of this boosted immunity against continually evolving viral genetic lineages.

**Conclusion & future perspective**

- To conclude, this review suggests that effective surveillance especially in immunocompromised patients and poorly vaccinated pockets of the world, equitable global distribution of vaccines, *in silico* and other antigenicity studies aimed at identification of conserved non-spike viral regions to design additional vaccines and therapeutics, technologies to tinker existing vaccines to neutralize future variants will help in preparedness for future preventive goals against SARS-CoV-2. The final aim, of course, would be to develop a ‘pan SARS-CoV-2 variant-proof’ vaccine to counter this monstrous virus.
